# A Personalized and Interactive Web-Based Advance Care Planning Intervention for Older Adults (Koda Health): Pilot Feasibility Study

**DOI:** 10.2196/54128

**Published:** 2024-05-06

**Authors:** R Lynae Roberts, Katelin D Cherry, Desh P Mohan, Tiffany Statler, Eric Kirkendall, Adam Moses, Jennifer McCraw, Andrew E Brown III, Tatiana Y Fofanova, Jennifer Gabbard

**Affiliations:** 1 Koda Health, Houston, TX, United States; 2 Atrium Health Wake Forest Baptist Medical Center, Winston-Salem, NC, United States; 3 Wake Forest Center for Healthcare Innovation, Winston-Salem, NC, United States; 4 Section of Gerontology and Geriatric Medicine, School of Medicine, Wake Forest University, Winston-Salem, NC, United States

**Keywords:** advance care planning, ACP, digital health tools, system usability, gerontology, geriatric, geriatrics, older adult, older adults, elder, elderly, older person, older people, ageing, aging, adoption, acceptance, usability, digital health, platform, website, websites

## Abstract

**Background:**

Advance care planning (ACP) is a process that involves patients expressing their personal goals, values, and future medical care preferences. Digital applications may help facilitate this process, though their use in older adults has not been adequately studied.

**Objective:**

This pilot study aimed to evaluate the reach, adoption, and usability of Koda Health, a web-based patient-facing ACP platform, among older adults.

**Methods:**

Older adults (aged 50 years and older) who had an active Epic MyChart account at an academic health care system in North Carolina were recruited to participate. A total of 2850 electronic invitations were sent through MyChart accounts with an embedded hyperlink to the Koda platform. Participants who agreed to participate were asked to complete pre- and posttest surveys before and after navigating through the Koda Health platform. Primary outcomes were reach, adoption, and System Usability Scale (SUS) scores. Exploratory outcomes included ACP knowledge and readiness.

**Results:**

A total of 161 participants enrolled in the study and created an account on the platform (age: mean 63, SD 9.3 years), with 80% (129/161) of these participants going on to complete all steps of the intervention, thereby generating an advance directive. Participants reported minimal difficulty in using the Koda platform, with an overall SUS score of 76.2. Additionally, knowledge of ACP (eg, mean increase from 3.2 to 4.2 on 5-point scale; *P*<.001) and readiness (eg, mean increase from 2.6 to 3.2 on readiness to discuss ACP with health care provider; *P*<.001) significantly increased from before to after the intervention.

**Conclusions:**

This study demonstrated that the Koda Health platform is feasible, had above-average usability, and improved ACP documentation of preferences in older adults. Our findings indicate that web-based health tools like Koda may help older individuals learn about and feel more comfortable with ACP while potentially facilitating greater engagement in care planning.

## Introduction

Advance care planning (ACP) is a process by which individuals choose their goals of care, quality of life priorities, and potential future medical intervention preferences and then communicate these values [1-3]. Actions taken during ACP include choosing a surrogate decision-maker (SDM), completing advance directives, and discussing a patient’s wishes with loved ones and health care providers. With increasing average lifespans, ACP is a vital component of high-quality care to ensure that patients’ care when facing serious illness is concordant with their values and goals. Currently, anywhere between 3% to 47% of patients may receive medical care that is not consistent with the patient or their loved ones’ wishes [4-6]. Because of these inconsistencies, it’s estimated that US $75.7 billion to US $101.2 billion is spent on overtreatment or low-value care each year in the United States [7]. These findings highlight the need to increase communication regarding medical care planning.

ACP is associated with decreased anxiety among patients’ family and caregivers [8], improved patient quality of life [9], decreased unwanted medical care [10-12], and decreased health care costs [13-16]. Despite the promising evidence of benefits [17], rates of ACP remain low, with many patients and families avoiding these discussions until the patient’s condition has deteriorated and is suboptimal for end-of-life decision-making. In addition, less than 11% of Medicare beneficiaries discuss ACP with their medical providers [18-21]. In the United States, approximately 37% or less of individuals report having some kind of advance directive, which could include a medical power of attorney or a living will [22,23]. ACP rates are often even lower within historically marginalized communities in America [24-28].

While the majority of patients express positive views or interest in ACP [22,29], they may not know how to begin the process. Initiation of ACP conversations seems to be a major barrier, as patients may be reluctant to broach the subject with their health care providers, while clinicians report having insufficient training or time to conduct ACP discussions [22,30,31] during busy clinic visits. Though patients believe that their medical providers should initiate conversations about ACP [32,33], 17% or fewer of patients report discussing ACP with their medical team [34]. Given the barriers for many patients in learning about and completing ACP, it is important to explore alternative approaches that may better support health care providers in facilitating this vital service [29]. Digital health tools could be a potential solution to improve equitable access to ACP for patients and to engage loved ones and health care providers in the process [35,36]. Scoping reviews have concluded that currently available web-based ACP programs are feasible and generally well-received by users [37,38], but the quality of the content greatly varies [39].

Current ACP online programs are primarily static web-based forms and do not include interactive educational content, plain language, or the capability to allow for official signing of ACP documentation [40]. Other ACP programs are geared toward specific patient populations and are therefore not generalizable to all individuals. Additionally, the sections on medical interventions in many online ACP resources fail to mention some common life support treatments that an individual may experience at end of life or with serious illness [41]. Several available smartphone apps also provide some education or actionable decision-making in regard to ACP, but currently available apps lack sufficient features or have poor functionality, limiting their practicality [42].

The Koda digital ACP platform seeks to fill the gaps found in current offerings by providing a highly interactive solution that is suitable for all technological knowledge levels and more inclusive of the most common life-support treatment options. The platform guides patients through a personalized, interactive guide, which includes video-based educational content and decision-making guides for patients and their loved ones. Users are able to select health care goals and indicate their wishes regarding potential future medical interventions, all of which auto-generate into easily accessible documentation that can be shared with loved ones and health care providers. Koda was created to help facilitate informed discussions of ACP and to provide a tool that patients could use freely and effectively without an added time burden for clinicians.

A previous retrospective report on Koda [43] was conducted with a sample of patients with serious illness. The findings of that quality improvement report showed that 53% of referred patients completed their ACP through the platform. However, due to the retrospective nature of this prior study, we were unable to assess self-report usability metrics or change in opinions or knowledge after platform use. The main objective of this study was to determine the reach, adoption, and usability of Koda to conduct digital ACP within a university health system.

## Methods

### Population and Recruitment

This pilot study included adult patients aged 50 years or older who had an active MyChart account, defined as one that was used within the past 90 days. Participants were excluded if they were younger than 50 years, were non–English speaking, had a diagnosis of Alzheimer disease or Alzheimer disease–related dementias, or had blindness based on electronic health record (EHR) *International Classification of Diseases, 10th Edition* codes.

An EHR algorithm was created to identify eligible participants (eg, filtering for the inclusion criteria) from the Atrium Health Wake Forest Baptist network (AHWFB). The EHR included data on age, gender, race, and diagnoses. The AHWFB is a large, quaternary health system affiliated with an accountable care organization program that incorporates more than 150 primary care and multispecialty practices with more than 330 physicians and advanced practice providers in 80 different locations in communities throughout central North Carolina.

Eligible participants were sent an electronic invitation to participate via Epic MyChart with an informational message about the study and an embedded hyperlink to the Koda platform. If a participant was interested, they were directed to a web-based consent form, and electronic informed consent was obtained. Participants were instructed to create a Koda Health account, complete a preassessment survey, complete using the Koda platform, and then complete a postassessment survey. Follow-up messages were sent 2 weeks later to any nonresponders. If a participant pressed “accept” but did not create a Koda account, they were approached 2 weeks later by the research team to facilitate the process.

### Ethical Considerations

This study was approved by the Atrium-Wake Forest Institutional Review Board (IRB00076779). All human subjects data were deidentified. Study participants were sent a US $25 gift card for completing the steps of the study.

### Intervention: Koda Digital Platform

**Figure 1.**
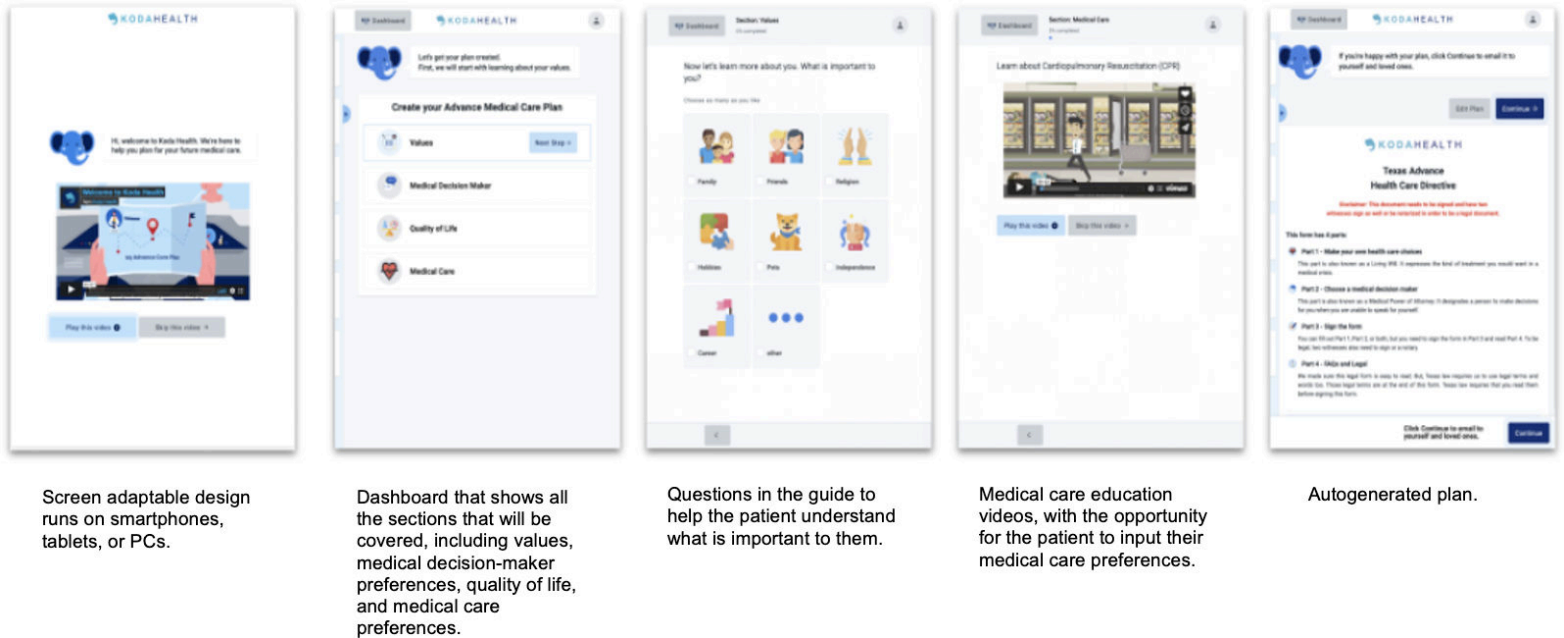
Overview of the Koda platform interface.

The Koda application was previously developed prior to this pilot study. Briefly, this occurred as an iterative process with input from ACP content experts (eg, geriatric and palliative medicine physicians), as well as input from end users surrounding their preferences, which was obtained by survey results. The patient-facing Koda ACP platform consists of 4 sections, focusing on values; individual definitions of quality of life; SDM preferences; and medical care preferences, with specific regard to cardiopulmonary resuscitation (CPR), mechanical ventilation, artificial nutrition via a feeding tube, and dialysis. This was in alignment with consensus recommendations on core components of ACP. Motivational interviewing techniques [44] were used to highlight the importance of planning and to motivate patients to communicate their wishes. Each section includes educational audio-video content and expandable information for additional questions users may have. For this study, platform completion progress was tracked through the Koda Health administration portal. Once the participant completed using the Koda platform by indicating their decisions within each section, their answers were autopopulated into a state-specific advance directive, which participants were able to sign or notarize online. Using the platform takes approximately 20-30 minutes. See Figure 1 for example displays directly from the platform.

### Outcome Measures

Primary and secondary outcome measures consisted of reach, adoption, and usability. Reach was defined as the proportion of eligible participants who clicked on the embedded hyperlink to the Koda application. Adoption was defined as the proportion of participants who completed using the Koda platform. Usability was measured using the validated System Usability Scale (SUS) [45,46], a 10-item questionnaire. Items are rated on a 5-point (ie, 0 to 4) scale and responses to all items are summed and multiplied by 2.5. Possible scores range from 0 to 100, with scores of 68 or higher indicating above-average usability [47]. The SUS was reliable for the current sample with α=.87, which is comparable to psychometric findings from multiple studies of the SUS (ranging from α=.83 to α=.97) [48].

Exploratory outcomes included ACP knowledge and readiness using an adapted, self-report ACP engagement survey [49] and were measured before and after using the Koda platform. To assess patients’ self-rated knowledge of ACP, 4 Likert-type questions were asked, which included “Do you know what Advance Care Planning is?” “How well informed are you about who can be a medical decision maker?” “How well informed are you about what makes a good medical decision maker?” and “How well informed are you about the different amounts of flexibility a person can give their medical decision maker?” The answer options were on a scale from 1 to 5, with higher scores correlating to higher ACP knowledge. Reliability for the adapted ACP Engagement Survey was good (α=.82) and comparable to previous psychometric research for other brief versions of this tool (ranging from α=.84 to α=.97) [49].

To assess patients’ readiness to engage with ACP, 4 Likert-type questions were asked, which included “What describes you best when it comes to your comfort level in thinking about your care if you become seriously ill?” “How ready are you to talk to your decision maker about the kind of medical care you would want if you were very sick or near the end of life?” and “How ready are you to talk to your healthcare provider about the kind of medical care you would want if you were very sick or near the end of life?” These answer options were on a scale from 1 to 5, with higher scores indicating greater readiness. The fourth question, “How worried are you when you think about your future illnesses?” was reverse scored so that higher scores would indicate less ACP readiness.

### Statistical Analyses

Reach, adoption, and SUS scores were calculated and reported as percentages and percentiles, respectively. Success was defined a priori by having ≥40% of enrolled participants complete using the Koda platform and obtaining an above-average SUS score of 68 or higher [50-52]. For descriptive analyses, means and SDs were used to describe continuous variables and percentages and frequencies were used to describe categorical variables. Shapiro-Wilk, D’Agostino-Pearson, and Anderson-Darling tests were conducted to assess data normality, and study variables were found to have non-Gaussian distribution. Therefore, nonparametric methods were used for any inferential analyses. Changes from pre- to post-Koda use were analyzed with the 2-tailed Wilcoxon signed rank test with continuity correction. Analyses were performed using R (version 4.2; R Foundation for Statistical Computing). *P*<.05 was considered to be statistically significant.

## Results

### Demographics

Table 1 shows participant demographic and baseline characteristics. The mean age was 62.8 (SD 9.3) years, with ages ranging from 50-99 years. Of enrolled participants, 127 (78.9%) were female, and 46 (28.6%) were Black or African American. A total of 49 patients (30.4%) self-reported poor to fair health, 55 (35.2%) had a personal loss or misfortune in the past year, and 103 (66%) had been a caregiver of a loved one with a serious illness.

**Table 1.** Demographics of participants who created a Koda account.

Characteristics		Participants (n=161)
Age (years), mean (SD)		62.8 (9.3)
**Sex*****,*** **n (%)**		
	Female	127 (78.9)
	Male	34 (21.1)
**Ethnicity, n (%)**		
	Hispanic or Latinx	1 (0.6)
	Not Hispanic or Latinx	159 (98.8)
	Not reported	1 (0.6)
**Race, n (%)**		
	American Indian/Alaska Native	1 (0.6)
	Black/African American	46 (28.6)
	White	113 (70.2)
**Self-reportd health status, n (%)**		
	Poor	10 (6.4)
	Fair	39 (25)
	Good	67 (42.9)
	Very good	36 (23.1)
	Excellent	4 (2.6)
**Personal loss/misfortune in last year, n (%)**		
	Yes, one	32 (20.5)
	Yes, more than one	23 (14.7)
	No	101 (64.7)
**Taken care of someone seriously ill, n (%)**		
	Yes	103 (66)
	No	53 (34)
**Trust in the health care system, n (%)**		
	Completely distrust	0 (0)
	Somewhat distrust	16 (9.9)
	Neither	23 (14.3)
	Somewhat trust	58 (36)
	Completely trust	14 (8.7)
	No response	50 (31.1)

### Reach, Adoption, and Usability of the Digital Koda Platform

**Figure 2.**
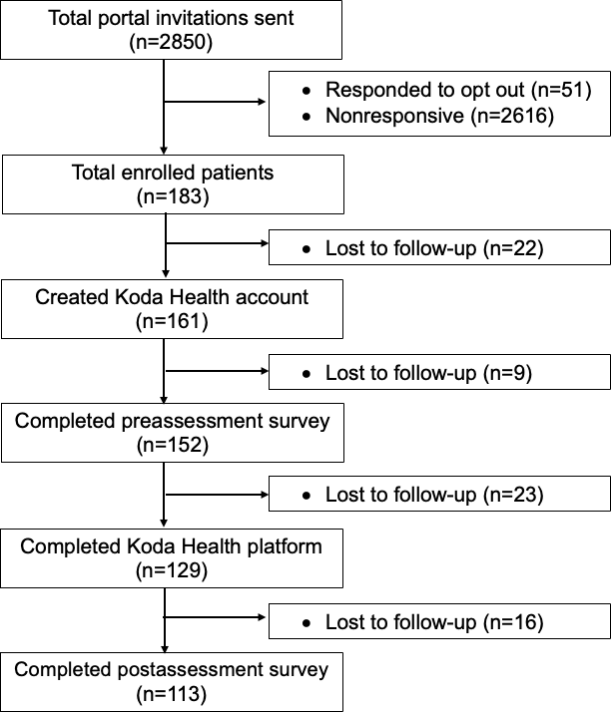
Flow diagram of participants through the study.

Of the 2850 patients who were sent the invitation to participate through their EHR patient portal, 183 participants read the message and clicked on the Koda link to enroll (6.4% response rate). Of those who responded to the invitation, 88% (n=161) created a Koda Health account to begin the study. Of the 161 participants who began their care plan on the platform, 129 (80.1%) completed the Koda platform intervention (Figure 2).

**Figure 3.**
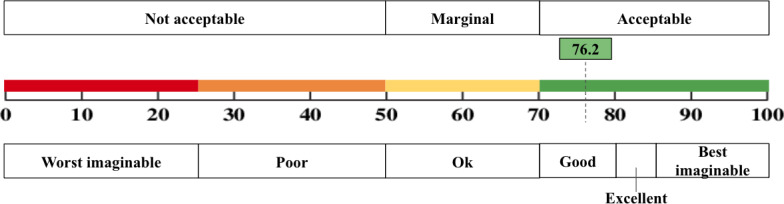
Demonstration of the acceptability and score range interpretations for the Koda platform. The score was 76.2 on the System Usability Scale, indicating that the platform was acceptable and had a good score.

As measured by the SUS, the participant-reported usability of the digital Koda platform was 76.2, indicating good system usability (Figure 3). Out of possible scores from 0 to 100, the SUS scores ranged from 47.5 to 100.

### Knowledge of ACP

Wilcoxon signed-rank tests showed that there was a significant increase in knowledge of ACP after using the Koda platform compared to baseline. Ratings increased on all 4 knowledge items: knowing what ACP is (*Z*=119.5; *P*<.001), knowing who can be an SDM *(Z*=202; *P*<.001), what makes a good SDM (*Z*=235; *P*<.001), and the amount of flexibility an SDM can have (*Z*=289; *P*<.001). Table 2 shows means at each time point.

**Table 2.** Change in knowledge of advance care planning (ACP) before and after completing the digital platform (on a 5-point scale).

Knowledge items	Before Koda use, mean rating (SD)	After Koda use, mean rating (SD)	*P* value
Knowing what ACP is	3.23 (1.31)	4.19 (0.97)	<.001
Knowing who can be an SDM^a^	3.63 (1.14)	4.28 (0.93)	<.001
Knowing what makes a good SDM	3.60 (1.16)	4.28 (0.90)	<.001
Knowing what flexibility an SDM can have	3.26 (1.29)	4.19 (0.96)	<.001

a SDM: surrogate decision-maker.

### Readiness to Make Decisions

Wilcoxon signed-rank tests showed that there was a statistically significant increase in readiness for ACP decisions after using the Koda platform compared to baseline. Ratings increased on 3 readiness items: comfort level thinking about serious illness care (*Z*=117; *P*<.001), readiness to discuss future medical care with an SDM (*Z*=316.5; *P*=.01), and readiness to discuss future medical care with a health care provider (*Z*=150; *P*<.001). The postassessment revealed a statistically significant decrease in ratings for how worried patients were about future illnesses (*Z*=536.5; *P*<.001). Table 3 shows means at each time point.

**Table 3.** Change in readiness for advance care planning (ACP) decisions and conversations before and after completing the digital platform (on a 5-point scale).

Readiness items	Before Koda use, mean rating (SD)	After Koda use, mean rating (SD)	*P* value
Comfort level thinking about serious illness care	2.89 (0.88)	3.06 (0.88)	<.001
Ready to discuss ACP with an SDM^a^	3.23 (1.46)	3.59 (1.32)	.007
Ready to discuss ACP with health care provider	2.78 (1.37)	3.26 (1.18)	<.001
Worried thinking about future illness^b^	3.32 (1.03)	3.07 (1.03)	<.001

a SDM: surrogate decision-maker.

b This item was reverse scored, so lower scores indicate higher ACP readiness.

## Discussion

This pilot study assessed the feasibility and acceptability of using a web-based interactive ACP platform, Koda Health, to help older adults think about their overall health-related goals, document those goals in an advance directive, and assign an SDM. We found that the intervention was acceptable to older adults and feasible to implement. This was highlighted by the fact that 80% of enrolled participants completed the entire intervention and created an advance directive.

Our findings also suggest that the Koda platform was able to bridge the gap in ACP engagement between different racial groups. Previous reports have shown that individuals from often-marginalized racial communities are less likely to have engaged in ACP [26,28]. However, when comparing the 2 main self-identified race categories in our data, we found no substantial difference—80.95% of Black patients and 79.66% of White patients who enrolled in the study went on to complete using the Koda platform. This adds to the existing literature that indicates that digital ACP platforms have the potential to address health disparities by providing accessible, user-friendly tools to all users, regardless of their racial background [43,53].

In addition, the SUS score of 76.2 indicates that users generally found the Koda platform easy to use and had a positive overall experience. This score suggests that Koda’s interface and features were well designed, allowing users to navigate and interact with the system without substantial barriers. We also found that patient age was not correlated with system usability ratings (*r*=.03; *P*=.76), suggesting that the platform was similarly user-friendly across the sample age range (50-99 years). However, while the overall usability was rated as good, there may still be room for improvement. It will be important to analyze usability metrics in more detail to identify specific areas where the platform can be enhanced. This could involve conducting further user testing, collecting qualitative feedback, or conducting additional surveys to gather more insights.

Participants also reported learning new information about ACP and being more ready to have conversations about medical care after completing the plan. While the self-reported changes in knowledge and readiness were statistically significant, we cannot yet accurately determine the degree of clinical significance. Generally, clinically significant improvements are associated with any positive increase to an average 5-point rating. However, more research is needed to determine specific thresholds for outcomes on ACP-related metrics [17,49]. Nevertheless, these findings have positive implications for the ability of online health tools to promote ACP participation among patients and families, with the ultimate goal of bringing empowerment and peace of mind during serious illness or end-of-life care.

Despite these promising findings, several limitations must be acknowledged. First, the study intervention necessitates further validation through a randomized controlled trial. Second, the low enrollment rate compared to the total number of invitees suggest that more effective recruitment methods are needed than a single patient portal message, such as personalized ACP information from a nurse or other health care provider, posters placed in prominent areas, or additional contact methods. Additionally, as in any voluntary research, responses may have been affected by self-selection bias; those who agreed to participate in the study may be distinct from those who chose to ignore the invitation to participate. We saw evidence of this in the greater percentage of female-identifying participants in the study than in the general population. Future studies should consider appropriate sampling techniques like stratified randomization to ensure participation reflective of the larger population. Lastly, further studies should investigate the long-term impacts of the Koda platform on measures of patient and caregiver experience and goal-concordant care [54].

In conclusion, the Koda ACP platform represents a promising tool for promoting patient engagement in ACP, particularly among older adults and marginalized groups. By facilitating knowledge acquisition and readiness to engage in ACP, the Koda platform can help empower patients to make goal-informed medical decisions, especially regarding end-of-life care. Further research is needed to validate these findings and determine long-term impacts on patient and caregiver outcomes.

## Abbreviations

ACP: advance care planning
AHWFB: Atrium Health Wake Forest Baptist
CPR: cardiopulmonary resuscitation
EHR: electronic health record
SDM: surrogate decision-maker
SUS: System Usability Scale
